# Intravenous and subcutaneous formulations of trastuzumab, and trastuzumab biosimilars: implications for clinical practice

**DOI:** 10.1038/s41416-020-01255-z

**Published:** 2021-02-16

**Authors:** Cornelius F. Waller, Julia Möbius, Adolfo Fuentes-Alburo

**Affiliations:** 1 Department of Haematology, Oncology and Stem Cell Transplantation, University Medical Centre Freiburg and Faculty of Medicine, University of Freiburg, Freiburg, Germany; 2 Mylan Healthcare GmbH, Hannover, Germany; 3 Mylan Inc., Canonsburg, PA USA

**Keywords:** Breast cancer, Chemotherapy

## Abstract

Trastuzumab is a biologic therapy indicated for the treatment of human epidermal growth factor receptor 2 (HER2)-positive breast cancer and metastatic gastric cancer. Trastuzumab was originally approved as an intravenous (IV) formulation but has since been developed for subcutaneous (SC) administration for patients with HER2-positive breast cancer. Both formulations demonstrate generally comparable pharmacological and clinical profiles. Therefore, when deciding between treatment options, factors such as the route of administration, patient preference, value and cost must be considered. Studies comparing IV with SC trastuzumab indicate that each formulation offers unique advantages to patients depending on their individual needs. Concurrent with the development of SC trastuzumab, IV trastuzumab biosimilars comprise another treatment option that, in view of their reduced cost, might improve patient access and increase cost-effectiveness for healthcare providers and payers. In this review, we seek to raise awareness of the current options available for trastuzumab so that healthcare providers can optimally treat patients according to their individual situations and preferences.

## Background

Between 15 and 20% of all breast cancers overexpress human epidermal growth factor receptor 2 (HER2).^1^ Over the past several decades, biologic therapies (in combination with chemotherapy) have emerged as paradigm-shifting treatments for patients with HER2-positive breast cancer, significantly improving patient outcomes and survival rates.^2^ Trastuzumab, a humanised anti-HER2 monoclonal antibody, is one such biologic therapy that is indicated for the treatment of HER2-positive metastatic breast cancer (MBC) and early breast cancer (EBC), as well as metastatic gastric cancer.^3,4^ Specifically, in the USA and the European Union, trastuzumab is approved for the treatment of HER2-positive MBC as first-line therapy in combination with paclitaxel, docetaxel or an aromatase inhibitor, and as second-line monotherapy. It is also approved for HER2-positive EBC as adjuvant therapy after surgery, chemotherapy or radiotherapy; in combination with paclitaxel or docetaxel, in combination with docetaxel and carboplatin; or in combination with neoadjuvant chemotherapy followed by adjuvant monotherapy.^3,4^

First approved in 1998 by the Food and Drug Administration (FDA) as an intravenous (IV) formulation, trastuzumab is administered as weekly infusions (initial dose of 4 mg/kg, then 2 mg/kg for 12 or 18 weeks, followed by maintenance doses of 6 mg/kg every 3 weeks [Q3W]) or infusions Q3W (initial dose of 8 mg/kg followed by maintenance doses of 6 mg/kg).^4^ A subcutaneous (SC) formulation of trastuzumab was subsequently approved in Europe in 2013 and in the USA in 2019.^5,6^ Currently, SC trastuzumab is indicated only for patients with HER2-positive breast cancer, administered Q3W as a fixed dose of 600 mg in a 5 ml volume.^6^ Both IV and SC formulations of trastuzumab continue to be used in the USA and Europe.

As a biologic therapy, trastuzumab can incur high costs to patients, which thereby potentially limits access for some patients and dissuades some healthcare providers from prescribing this agent. However, the increasing availability of biosimilars is expected to substantially reduce costs associated with biologics over the next decade,^7^ although the availability and uptake of biosimilars could vary by region; depend on a variety of country-specific factors; and be influenced by the needs of healthcare systems, policymakers and payers.^8^ Biosimilars are highly similar to their reference product in terms of quality, efficacy, safety and immunogenicity, and must meet the same standards as the reference biologics,^9,10^ but usually cost less than the reference product, as they generally have lower development costs compared with their reference biologics. Patent expirations for trastuzumab in the European Union (2014) and USA (2019) have prompted the development of several trastuzumab biosimilars^11^ and, to date, five trastuzumab biosimilars have been approved by the FDA and European Medicines Agency: trastuzumab-dkst (Mylan GmbH, Steinhausen, Switzerland), trastuzumab-pkrb (Celltrion Inc., Incheon, Republic of Korea), trastuzumab-dttb (Samsung Bioepis Co., Ltd, Incheon, Republic of Korea), trastuzumab-qyyp (Pfizer Ireland Pharmaceuticals, Cork, Ireland) and trastuzumab-anns (Amgen Inc., Thousand Oaks, CA, USA).^11,12^ All currently approved trastuzumab biosimilars are formulated for IV use only, although the eventual patent expiration of SC trastuzumab (anticipated in 2030 in the USA) might allow for the development of SC biosimilar formulations in the future.

The FDA approval of the trastuzumab SC formulation has led to increasing interest in understanding the efficacy and safety profiles of IV and SC trastuzumab. Furthermore, the emergence of trastuzumab biosimilars, although initially available in IV formulation only, adds an additional layer to the healthcare provider decision-making process. This review summarises the pharmacokinetic, efficacy and safety profiles of IV and SC trastuzumab formulations before discussing additional factors—including route of administration and resource utilisation factors—involved in the use of these formulations and in the use of biosimilars, which might influence patient and healthcare provider preferences and, ultimately, treatment decisions.

## Pharmacological and clinical comparison of IV versus SC trastuzumab

Several studies have examined the dosing of SC trastuzumab required to produce pharmacokinetic exposures and serum trough concentrations (C_trough_) similar to those of IV trastuzumab.^13,14^ Additionally, several studies have compared the efficacy and safety of IV versus SC trastuzumab.^15–18^ These key studies have demonstrated that the overall pharmacological and clinical profiles of SC and IV trastuzumab are comparable, and that both formulations are safe and effective therapies for patients with HER2-positive MBC and EBC. Data from the comparative studies are summarised in Table 1, and specific findings are detailed below.

**Table 1:** Pharmacokinetic, efficacy, and safety data from key trials comparing IV and SC trastuzumab formulations.

	IV trastuzumab	SC trastuzumab
Indication	HER2-positive MBC, HER2-positive EBC, HER2-positive metastatic gastric cancer	HER2-positive MBC, HER2-positive EBC
Reconstitution	Bacteriostatic water (420 mg/20 ml)	Hyaluronidase solution (5 ml)
Dose	4 mg/kg (loading), 2 mg/kg weekly (12 or 18 weeks), 6 mg/kg Q3W (maintenance); 8 mg/kg (loading), 6 mg/kg Q3W (maintenance)	600 mg Q3W
Initial approval	US: 1998; EU: 2000	US: 2019; EU: 2013
Phase 1/1b (NCT00800436)^12^		
Pharmacokinetics		
AUC (μg·d/ml)	1800	2090
Terminal half-life (d)	10	10
HannaH trial (NCT00950300)^14,15^		
Pharmacokinetics		
C_trough_ (pre-dose) levels >20 μg/ml (%)	98.7	97.0
Efficacy		
Pathological complete response (%)	40.7	45.4
Overall response rate (%)^a^	88.8	87.2
Median time to response (wk)	6.0	6.0
6-y event-free survival (%)	65.0	65.0
6-y overall survival (%)	84.0	84.0
Safety		
≥1 AE (%)	93.9	97.3
≥1 Serious AE (%)	12.4	20.9
Antidrug antibodies (%)	3.4	6.8
PrefHer trial (NCT01401166)^16^		
Safety		
AEs, switching SC → IV (%)	48.7	65.4
AEs, switching IV → SC (%)	53.8	56.4
MetaspHer trial (NCT01810393)^17^		
Safety		
AEs (%)	44.1	67.6

*AE* adverse event, *AUC* area under the curve, *EBC* early breast cancer, *EU* European Union, *HER* human epidermal growth factor receptor, *IV* intravenous, *MBC* metastatic breast cancer, *Q3W* every 3 weeks, *SC* subcutaneous, *US* United States.

^a^Only patients with measurable disease at baseline were included.

### Pharmacokinetics

In an open-label, Phase 1/1b, two-part, dose-finding study in healthy male volunteers and HER2-positive female patients with EBC, SC trastuzumab (8 mg/kg) attained pharmacokinetic values and exposures comparable with those of IV trastuzumab (6 mg/kg).^13^ The terminal half-life for both IV and SC trastuzumab administrations was ~10 days.^13^ On the basis of these data and population pharmacokinetic modelling, a fixed dose of SC trastuzumab 600 mg given Q3W was shown to produce a C_trough_ and an exposure equivalent to those of the IV formulation.^13,19^

The open-label, Phase 3 Enhanced Treatment With Neoadjuvant Herceptin (HannaH) trial evaluated the effect of IV trastuzumab and fixed-dose SC trastuzumab on C_trough_ in patients with HER2-positive EBC at pre-dose cycle 8 before surgery and pathological complete response (pCR) in the neoadjuvant setting.^15^ The proportion of patients with C_trough_ (pre-dose) levels >20 μg/ml was high and similar for the IV (98.7%) and SC dose groups (97.0%) before cycle 8. After a median follow-up of ~12 months, the pharmacokinetic profile of the fixed 600 mg SC dose was noninferior to that of the standard IV dose with a geometric mean C_trough_ ratio (C_trough_ SC to C_trough_ IV) of 1.33.

The possible effect of body weight on the efficacy of IV and SC formulations might, however, require additional evaluation. A subgroup analysis of the HannaH trial suggested that patients with body weight >80 kg might experience reduced efficacy with the SC formulation compared with the IV formulation, but these results did not reach statistical significance.^20^ Furthermore, preliminary data from a small observational trial in patients with HER2-positive non-MBC indicated that, in obese patients, a fixed-dose SC regimen of 600 mg was not equivalent to a dosage regimen adjusted to the patient’s body weight at a dose of 6 mg/kg IV.^21^ Additional research, including larger controlled trials, is therefore required to understand the clinical implication(s) of these findings.

### Efficacy

In combination with chemotherapy, IV trastuzumab (and its biosimilars) has robustly been shown to improve overall survival and overall response rates in patients with MBC.^22–26^ In the Phase 3 HannaH trial, comparable efficacy was observed between IV and SC formulations of trastuzumab in patients with HER2-positive EBC.^15,16^ In the neoadjuvant setting, SC trastuzumab was noninferior to IV trastuzumab with respect to pCR (45.4% and 40.7%, for SC and IV, respectively); the difference in pCR between groups (4.7%) was not statistically significant.^15^ Overall response (defined as clinical complete response or partial tumour response) was also similar between the IV (88.8%) and SC (87.2%) formulations, and the median time to response was 6 weeks for both groups.^15^ The 6-year event-free survival rate was 65% and the 6-year overall survival rate was 84% in both groups.^16^ Overall, results from the open-label HannaH trial demonstrated no clinically meaningful differences in efficacy between IV and SC trastuzumab formulations, supporting the comparable efficacy profile of both formulations.

### Safety

The safety profiles of IV and SC formulations of trastuzumab have been individually studied in separate clinical trials.^23,27,28^ In Phase 2 and Phase 3 studies evaluating IV trastuzumab in patients with HER2-positive breast cancer, the most commonly reported adverse events (AEs) were infections, headache, nausea, fever and chills.^4,23,27^ In the SafeHer trial, a two-cohort, nonrandomised open-label study of the overall safety of SC trastuzumab in combination with chemotherapy in patients with HER2-positive breast cancer, the most commonly reported AEs were diarrhoea, fatigue and arthralgia.^28^

In addition to being studied in separate trials, the safety profiles of IV and SC trastuzumab have also been compared within the same trial. In the open-label Phase 3 HannaH trial, no difference in the incidence of AEs was observed between IV (93.9% [280/298]) and SC trastuzumab (97.3% [289/297]) in patients with HER2-positive EBC, although more patients in the SC group than in the IV group had AEs that were classed as serious (20.9% [62/297] versus 12.4% [37/298], respectively). This difference in incidence of serious AEs was partly attributable to infections and infestations (SC 8.1% versus IV 4.4%), while the incidence of other serious AEs was spread across system organ classes; the most common serious AEs occurring in two or more patients included febrile neutropenia (SC 4.4%; IV 3.4%) and neutropenia (SC 2.4%; IV 3.0%).^15^ The most common AEs in both groups were alopecia, nausea, neutropenia, diarrhoea, asthenia and fatigue. Cardiac safety profiles were comparable between the groups. When safety was evaluated after 6 years, the incidence of AEs remained similar between those receiving IV (94.6% [282/298]) and SC (97.6% [290/297]) trastuzumab.^16^

The immunogenicity of trastuzumab was examined in the open-label Phase 3 HannaH trial. For either the IV or SC trastuzumab formulation, few patients were positive for antidrug antibodies post baseline; of these few patients, however, twice as many patients receiving the SC formulation developed post baseline antidrug antibodies to trastuzumab compared with those receiving the IV formulation (6.8% [20/295] versus 3.4% [10/295], respectively).^15^ Over 11% (34/295) of patients receiving SC trastuzumab had post baseline anti-recombinant human hyaluronidase PH-20 antibodies, consistent with the addition of recombinant human hyaluronidase to the SC formulation for improved dispersal and absorption.^6^ Neutralising antibodies were not detected in either group, and antidrug antibodies did not affect efficacy or infusion-related reactions.

Along with individual patient needs, it is important to understand safety considerations that might arise when switching between formulations. For example, healthcare providers might identify a need to change between formulations over the course of an individual patient’s treatment, perhaps because of patient preference or AEs associated with the initial route of administration. This issue was assessed in the PrefHer trial, a crossover study evaluating safety and patient preference with adjuvant SC versus IV administration of trastuzumab after completion of neoadjuvant chemotherapy in patients with HER2-positive EBC.^17,29^ In this trial, patients switched from four cycles of SC trastuzumab to four cycles of IV trastuzumab, and vice versa.^17^ In patients switching from SC to IV, the rates of AEs were 65.4% (159/243) and 48.7% (116/238), respectively. In patients switching from IV to SC, rates of AEs were 53.8% (129/240) and 56.4% (133/236), respectively. Incidences of grade ≥3 AEs, serious AEs, AEs leading to study drug discontinuation and cardiac AEs were similar between formulations.^17^ Overall, no new safety signals emerged after switching formulations in either direction.

Similar to PrefHer, the Phase 3 MetaspHer trial is a crossover study that compared safety and patient preference when switching between IV and SC administration of trastuzumab (and vice versa) in patients with MBC.^18^ In this trial, more AEs were observed in patients receiving SC trastuzumab (67.6% [73/108]) than those receiving IV trastuzumab (44.1% [49/111]), regardless of treatment sequence, and the most frequent AEs were asthenia, fatigue, injection site erythema and pain, infections and infestations, arthralgia, muscle spasms and headache. Overall, however, SC trastuzumab was generally well tolerated and no new safety signals emerged relative to the known safety signals of the IV formulation and SC formulation at the early stage.

One aspect that was not prospectively evaluated in these trials comparing preference for IV versus SC formulation was the potential pain associated with the 5 ml injection volume of the SC formulation. Subcutaneous injections of ≥1.5 ml are thought to be associated with pain and injection site reactions, although no formal evidence supports this notion.^30^ However, if required for the drug to be efficacious, injection volumes that are ≥3.5 ml merit further exploration regarding potential inflicted pain and injection site reactions.^30^ Additionally, it is the experience of the authors in clinical practice that some patients experience discomfort with SC injection but might not wish to discuss this discomfort with their healthcare provider. However, further assessments are needed to understand the frequency with which healthcare providers discuss this topic with their patients as well as the impact of potential pain on patient preference for SC formulations.

## Additional considerations for therapeutic options

Given the similar pharmacokinetic and clinical profiles of the IV and SC trastuzumab formulations, a number of additional factors, such as route of administration, patient preference, value of the health technology and cost, should be considered by healthcare providers during their decision making regarding which formulation of trastuzumab to administer. Additionally, given the increasing availability of trastuzumab biosimilars,^11,12^ healthcare providers should contemplate the possible advantages of these agents, too, when evaluating potential biologic therapeutic options. The current biologic therapy landscape of EBC and MBC is rich, and offers numerous options for patients and healthcare providers, which necessitates a careful consideration of preferences and costs involved, as outlined below.

### Route of administration

In patients with HER2-positive EBC or MBC who are receiving trastuzumab as monotherapy, SC administration can offer a time-saving option owing to reported shorter administration times.^31^ According to the prescribing information, IV trastuzumab requires an initial 90-min infusion, and subsequent infusions require between 30 and 90 min,^4^ whereas administration of SC trastuzumab requires ~2–5 min.^6^ However, as healthcare providers can treat multiple patients receiving IV infusions simultaneously, the apparent time savings conferred by SC outlined in the literature might be overestimated.^14^ Follow-up observations of similar duration for both IV and SC formulations (6 h after the first administration and 2 h after each of the following administrations) are recommended.^32^ A time-and-motion study alongside the PrefHer clinical trial evaluated the length of time for administration of SC compared with IV trastuzumab.^33^ The mean amount of healthcare provider time required for one SC administration of trastuzumab was 24.6 min compared with 58.1 min for an IV administration. On average, patients receiving IV trastuzumab spent more time in the healthcare unit (94.5 versus 30.3 min) and had a longer mean chair time (75.0 versus 19.8 min) compared with those receiving SC trastuzumab.

A 2015 publication evaluated real-world experiences with IV and SC trastuzumab in patients with HER2-positive MBC (20–180 patients per centre) or EBC (100–600 patients per centre) in seven German healthcare centres over 18 months.^31^ This report of practical experience demonstrated that the SC formulation was associated with greater appointment flexibility for patients and healthcare centres, but uncovered no significant difference between IV and SC formulations in terms of cumulative workload and healthcare provider time associated with administration. The results also suggest that in settings where trastuzumab is administered to patients with HER2-positive MBC or EBC in combination with another IV therapy (e.g. chemotherapy, pertuzumab), the IV route might be chosen because a central venous port is already in place.

### Patient preference

IV administration of trastuzumab might be preferred by patients who are receiving chemotherapy or other IV therapies as they already have a central venous port in place and the ability to avoid an additional injection might be preferred over potential time savings.^31^ However, this view might not be shared by all patients. In a retrospective cohort analysis of Swedish patients with EBC receiving IV trastuzumab, 34 out of 35 patients (97.4%) chose to switch to SC trastuzumab despite most of these patients receiving an initial combination with IV chemotherapy; the study also found no evidence that the initial IV formulation was chosen over the SC formulation during chemotherapy.^34^ It is important to note, however, that this study excluded patients with MBC, thereby limiting comparisons of IV and SC trastuzumab across different disease stages.

The open-label PrefHer (EBC) and MetaspHer (MBC) trials also examined preference between trastuzumab formulations.^18,29^ In these trials, patients who switched from IV to SC trastuzumab and vice versa were asked about their preference after the crossover period. Of the 467 patients in the PrefHer trial, 224 (48.0%) had received prior IV therapy via cannula, 206 (44.1%) had received prior IV therapy via venous access device and 37 (7.9%) had received prior IV therapy via both methods. Regardless of switch from IV to SC or vice versa, a majority of patients (415; 88.9%) reported that they would choose SC over IV administration; this proportion was significantly greater than the predicted preference rate of 65% (*P* < 0.0001) that was assumed by the investigators.^29,35^ IV administration was the preferred choice of 45 (9.6%) patients, and 7 (1.5%) had no preference. Patients reportedly preferred SC administration because of time saved, decreased pain and discomfort, or fewer side effects. Conversely, patients reportedly preferred IV administration because of fewer injection site reactions than SC administration, more efficient use of their IV port, and more time to ‘settle’ into the treatment setting and interact with nurses and other patients.^29^ In the MetaspHer trial, patients with MBC also reported a preference for SC over IV administration; overall, 85.9% (79/92) of patients preferred SC administration compared with 14.1% (13/92) preferring IV administration (*P* < 0.001),^18^ but no specific reasons for preference were recorded.

### Costs associated with SC versus IV trastuzumab

Several time-and-motion studies have been undertaken to estimate the differences in cost between IV and SC administration. In a UK-based time-and-motion substudy of the PrefHer clinical trial, derived costs for healthcare provider time and consumables per IV treatment were £132.05 and £12.92, respectively, compared with £31.99 and £1.17 per SC treatment, respectively, resulting in a total difference of £111.81 between formulations per treatment.^33^ These data suggest that over a full treatment course (i.e. 18 cycles), treatment with SC trastuzumab might be associated with cost savings of over £2000 per patient compared with IV administration. In a Belgian time-and-motion study, IV administration costs for healthcare provider time and consumables were €50.40 more than for SC administration at each treatment visit.^36^ However, this analysis did not compare resource differences between MBC and EBC (e.g. resources that might be associated with the care of patients with varying disease severity and possible associated complications and comorbidities). Furthermore, both studies could be limited as they might not reflect typical clinical practice and did not consider resource utilisation in patients with IV catheters (e.g. resources associated with the insertion and maintenance of catheters or resources needed to address potential complications, such as infections and thrombosis, that might occur with implanted catheters).^33,36^

Interestingly, another publication listed higher drug cost for the IV formulation than for the SC formulation but did not differentiate sufficiently between cost factors.^37^

### Costs associated with trastuzumab biosimilars

Although several studies have estimated potential healthcare utilisation and cost savings associated with SC administration of trastuzumab in comparison with IV administration, such analyses have predominantly overlooked the possible cost benefits of IV trastuzumab biosimilars, which began entering the market after the reference product patent expirations in 2014 (EU) and 2019 (USA),^11,14,34^ and thereby limit a comprehensive assessment of economic benefits. Biosimilars might offer substantial drug-related cost savings, with discounts, generally, of ~30%^7^ but possibly as high as 69%,^38^ over reference products, challenging the cost advantages associated with SC trastuzumab. Despite the biosimilars market still being in its infancy, it is estimated that these agents could reduce the overall costs associated with biologics by $54 billion between 2017 and 2026 in the USA.^7^ Hospital-based, regional and national tendering policies, as well as pharmacist-led substitution (if permitted), might promote the use of biosimilar medicines.^39,40^ Furthermore, regional guidance might incentivise physicians to prescribe biosimilars over reference biologics: while some countries (e.g. Belgium, Germany) encourage healthcare providers to prescribe biosimilars for a certain minimum number of treatment-naive patients, other countries (e.g. Austria, Norway) call upon healthcare providers to prescribe the most cost-effective or cheapest treatment option, which is often a biosimilar, unless a valid clinical reason is provided.^38^

Two analyses have evaluated the potential cost savings associated with using biosimilar trastuzumab over reference trastuzumab. One reported the potential financial impact of biosimilar trastuzumab in trastuzumab-naive Croatian patients with either MBC or EBC, assuming a biosimilar uptake of 50%. The projected drug cost savings ranged from €0.26 million to €0.69 million (assuming a price discount of 15% and 35%, respectively) after 1 year.^41^ The other budget impact analysis modelled the financial effects of using biosimilar trastuzumab in 28 European countries and in patients with HER2-positive EBC, MBC or metastatic gastric cancer.^42^ Assuming a 30% discount, a 20% switching rate in the first year and a 5% switching rate in the following years (estimated market uptake assumptions similar to the real biosimilar market share reported in European countries), estimated savings within the first year ranged from €58 million to €68 million. Over 5 years, potential savings ranged from €0.91 billion to €2.27 billion, depending on the scenario, which could allow ~55,000 to ~116,000 more patients to be treated with biosimilar trastuzumab.

Importantly, because of their reduced costs, biosimilars might open up new treatment avenues for patients who previously could not afford biologic therapy,^7,11^ thereby representing additional ‘value’.^43^ The availability of biosimilars might also drive down the costs of reference products or the entire product class.^11^ The high cost of biologics, including reference trastuzumab, can be a barrier to patient access, and healthcare providers can be deterred from prescribing biologic therapies because of their high cost, despite their favourable efficacy. The availability of biosimilars could change this perspective, particularly for the treatment of breast cancer. For instance, a 2014 survey reported that nearly half of oncologists would increase the prescription of anti-HER2 biologics across multiple settings if a lower-cost biosimilar was available.^44^ The cost savings and improved patient access associated with biosimilars are largely dependent on physician awareness of, and access to, these life-saving medications.^11^ Accordingly, future cost-effectiveness analyses should examine biosimilars, including biosimilars of trastuzumab, and increase awareness among healthcare providers of these newly emerging and potentially cost-saving therapies.

## Conclusions

The anti-HER2 monoclonal antibody trastuzumab is indicated for the treatment of HER2-positive breast cancer and metastatic gastric cancer. Trastuzumab is available in both IV and SC formulations (SC is for breast cancer only), and the pharmacological and clinical profiles of both formulations have been found to be comparable across several trials. Given the similarity in pharmacokinetics, efficacy and safety, other factors—such as patient preference, value to patients and the healthcare system, and cost—might therefore be considered when choosing between these therapeutic formulations. Biosimilars, which are only available in IV formulation due to existing patents, provide another important treatment consideration because of potential cost-effectiveness and improved patient access.

The advantages of SC trastuzumab include potentially shorter administration times, although the post-observation times for both formulations are identical. Open-label patient preference studies have suggested that a majority preferred the SC route of administration; however, some patients might prefer IV administration because trastuzumab is often administered in combination with other IV therapies, including chemotherapy and pertuzumab, another anti-HER2 antibody. For these patients, IV trastuzumab might be more practical to avoid additional injections. Additionally, patients who have received previous therapy retain their IV ports for an extended period of time even after chemotherapy has ended; the presence of this port might influence the decision of IV versus SC administration.

IV trastuzumab biosimilars provide comparable clinical benefits to IV trastuzumab and the patent-protected SC trastuzumab formulation but might also offer value to patients and healthcare providers as a more cost-effective treatment option and could increase patient access to otherwise expensive biologic therapies. Given the general reduction in cost associated with the introduction of a biosimilar to the market, it is unlikely that the reduced administration costs associated with the SC formulation of trastuzumab will outweigh the lower cost of IV biosimilars.^11^ It is therefore important for healthcare providers to consider the potential benefits of IV trastuzumab biosimilars (e.g. lack of need for additional injections) and whether they outweigh the benefits associated with SC administration, as well as to consider patient preferences, on individual bases, regarding the route of administration.

In conclusion, trastuzumab has long been a cornerstone of therapy for the treatment of HER2-positive breast cancer and metastatic gastric cancer. Healthcare providers, patients and caregivers must work together to decide which formulation and route of administration best meets their needs, particularly as additional data (e.g. efficacy and safety of the small molecule tyrosine kinase inhibitor tucatinib plus trastuzumab in HER2-positive MBC) become available and potentially expand the array of treatment options. Additionally, biosimilars should be included in future cost-effectiveness analyses and models; increasing access to this information will help healthcare providers to better understand the potential cost savings and encourage them to make informed decisions that will benefit their patients. Importantly, patient perceptions of other formulations in development for HER2-positive EBC (e.g. an SC fixed-dose combination of pertuzumab and trastuzumab administered in a single injection), and how patients and healthcare providers weigh these options against the financial advantages of biosimilar trastuzumab (and, in the future, biosimilar pertuzumab), remain to be seen.

## Data Availability

Not applicable.
