# Correction to: Intravenous and subcutaneous formulations of trastuzumab, and trastuzumab biosimilars: implications for clinical practice

**DOI:** 10.1038/s41416-024-02769-6

**Published:** 2024-07-03

**Authors:** Cornelius F. Waller, Julia Möbius, Adolfo Fuentes-Alburo

**Affiliations:** 1 Department of Haematology, Oncology and Stem Cell Transplantation, University Medical Centre Freiburg and Faculty of Medicine, University of Freiburg, Freiburg, Germany; 2 Mylan Healthcare GmbH, Hannover, Germany; 3 Mylan Inc., Canonsburg, PA USA

Correction to: *British Journal of Cancer* 10.1038/s41416-020-01255-z, published online 16 February 2021

After publication of our article [1] we became aware that the Competing Interests statement was incomplete. It should read as follows: “C.F.W. is a consultant/advisory board member for Mylan Inc. A.F.-A. is a paid employee of Mylan Inc. and may hold stock with the company. J.M. is a paid employee of Mylan Healthcare GmbH in Hannover, Germany, and may hold stock with the company. Mylan Inc. produces trastuzumab for intravenous administration.”

The original article has been corrected.
